# Gender Differences in the Clinical Presentation of Cluster Headache: A Role for Sexual Hormones?

**DOI:** 10.3389/fneur.2019.01220

**Published:** 2019-11-22

**Authors:** Marta Allena, Roberto De Icco, Grazia Sances, Lara Ahmad, Alessia Putortì, Ennio Pucci, Rosaria Greco, Cristina Tassorelli

**Affiliations:** ^1^Headache Science Centre, IRCCS Mondino Foundation, Pavia, Italy; ^2^Department of Brain and Behavioral Sciences, University of Pavia, Pavia, Italy

**Keywords:** cluster headache, female, sex-differences, gender-related variables, migraine

## Abstract

**Introduction:** Cluster Headache (CH) is a well-characterized primary headache that mostly affects men, although a progressive decrease in the male-to-female ratio has occurred over time. Available, but partly discordant, data on gender-related differences in CH suggest a more marked overlapping with migraine features in female subjects. The aim of this study is to carefully evaluate the female/male distribution of the typical migraine-associated symptoms and of other features of the disease in a large and well-characterized clinical population of CH subjects.

**Materials and Methods:** We enrolled consecutive CH patients regularly followed at the tertiary Headache Science Center of the IRCCS Mondino Foundation of Pavia (Italy) who attended the Center for a CH bout between September 2016 and October 2018. The subjects were requested to fill in a semi-structured questionnaire focused on the presence of migraine-associated symptoms, familiarity for migraine and, for women, the relationship of CH onset with the reproductive events of their life. These data were compared and integrated with those recorded over time in our clinical database, including demographics and clinical characteristics. The primary outcome was the gender distribution of subjects who satisfied ICHD-III criterion D for migraine-associated symptoms. The secondary outcomes were represented by the gender distribution of individual migraine-associated symptoms and of other disease features included in the questionnaire and/or in the clinical database.

**Results:** Data from 163 males (mean age 41.46 ± 10.37) and 87 females suffering of CH (mean age 42.24 ± 11.95) were analyzed. We did not find a different distribution between sexes as regards the primary outcome measure (F 73.6%, M 65.6%, *p* = 0.200). However, when we analyzed the occurrence of individual symptoms, nausea and osmophobia were reported more frequently by women (*p* = 0.048, *p* = 0.037, respectively). Ptosis and nasal congestion were predominant in females (*p* = 0.017 and *p* = 0.01, respectively), while enlarged temporal artery was more frequently reported by men (*p* = 0.001). Distribution of pain across the head tended to be larger in women, extending more frequently to the zygomatic (*p* = 0.050), parietal (*p* = 0.049), and frontal (*p* = 0.037) regions. Women had a longer mean attack duration (*p* = 0.004) than men. In CH women the onset of disease often corresponded with moments of important changes in the levels of sexual hormones (menarche, post-partum, menopause). Concomitant thyroid diseases and psychiatric disorders were observed more frequently in women than in men, while snoring and smoking habit was reported by a higher percentage of men than women.

**Conclusion:** We confirmed the presence of distinct gender-related differences in CH and added some novel information that lends credibility to the hypothesis of a closer phenotypical similarity between CH and migraine in the female sex. These observations are relevant for advancing our knowledge on CH pathophysiology, as well as for a more refined diagnostic framing and improved management of the disease.

## Introduction

Cluster Headache (CH) is a rare but phenotypically well-characterized primary headache disorder. According to the diagnostic criteria defined by the International Classification of Headache Disorders (ICHD-III) (1) CH is a strictly unilateral headache occurring in attacks lasting 15–180 min and characterized by very severe pain commonly localized in the orbital or sovraorbital area, associated to ipsilateral autonomic symptom (ptosis or miosis, lacrimation, eyelid oedema, sweating, conjunctival injection, lacrimation, nasal congestion, rhinorrhea) or a sense of restlessness, or both. CH exists in two forms: the episodic one being the most prevalent, and the chronic one, more rare, which may evolve from the episodic or less frequently start *de novo*.

Originally considered a male disorder, CH has been described more and more frequently in women. The data from the literature have indeed pointed to a progressive reduction in the male-to-female ratio over the years, with a transition from the initial 5–7:1 reported 40 years ago (2, 3), to the more recent 2–3:1 (4, 5). The reasons behind the ratio modification are not clear but several factors have been proposed. *In primis*, an improvement in the diagnostic accuracy, which has led to the correct diagnosis of CH in women previously misdiagnosed as migraine sufferers (4). Another possible explanation is represented by the profound changes occurred in our society in the last decades leading to the redistribution among sexes of environmental and life habit factors likely to play an etiological role in CH, e.g., stress, alcohol and smoking habit, etc. (6).

Although CH attacks are very clear-cut, studies over the years have revealed gender differences (7). In a retrospective study, Rozen et al. (8) reported an increased occurrence of nausea and vomiting in CH women, a finding that has been recently confirmed in an large internet survey (9). Manzoni et al. reported an increased occurrence of nausea but not of vomiting in CH females (10). On the contrary, Dong et al. failed to detect any difference between sexes in a relatively quite large clinical population of CH subjects, which however included a small number of women (11). CH men seem more likely to have cranial autonomic symptoms (9, 12), although these seem to be less pronounced in the subjects who experience a late onset of the disease (12). In contrast with these findings, a larger Danish survey has reported an increased occurrence of ptosis, eyelid edema in CH women when compared to men (4). Interestingly, episodic CH shows a bimodal distribution of age onset in women, with the second peak occurring around the menopause (13, 14). Other studies have suggested an association between cluster headache and hormonal fluctuations, with the report of more severe CH attacks during the menstrual period, a tendency toward the improvement during pregnancy and a possible negative effect of oral contraception and hormonal replacement therapy (5, 15, 16). Finally, CH women tend to have a positive family history of migraine more frequently than CH men (2, 10).

Though intriguing, all these findings remain so far inconclusive, hence the need to further investigate gender-related differences in CH. The primary aim of this study was to focus on the occurrence during CH attacks of migraine-associated symptoms—strictly defined according to ICHD-III criterion D for migraine without aura—in a representative population of CH subjects regularly followed at our Headache Center.

## Materials and Methods

We conducted a cross-sectional evaluation of the consecutive CH subjects regularly followed at the tertiary Headache Science Center of the IRCSS Mondino Foundation of Pavia (Italy) who attended the Center for a CH bout in the period between September 2016 and October 2018. The study was evaluated and approved by our local Ethics Committee (which in 2016 was held jointly with San Raffaele Scientific Institute—Milan, Italy).

During the visit, which was performed by a neurologist with a long expertise in headache, the patients' diagnosis was confirmed against the ICHD-III criteria for CH. After signing the informed consent for the study, patients were asked to fill in a semi-structured questionnaire specifically devised for the study. The questionnaire focused on the presence of migraine-associated symptoms (nausea, vomiting, phonofobia, photophobia, and osmophobia), familiarity for migraine and, for women, relationship of CH onset with reproductive events (menarche, menstrual cycle, duration of periods, use of contraceptive pills, number of pregnancies, menopause).

During the visit we also collected data regarding the characteristics of the attacks (frequency, duration, severity, associated symptoms, response to acute treatment) and of the most recent bouts (frequency, duration, response to preventive treatments). These latter pieces of information were compared with the data available from the same patients in our clinical database in order to minimize recall biases. Our clinical database indeed is continuously updated at each patient visit and contains general demographics (age, sex, occupation, lifestyle factors), information regarding cluster headache type, characteristics and recurrence of attacks (location, severity, duration, and frequency of pain, associated autonomic symptoms, associated migraine-like symptoms, circadian and circannual frequency, duration), acute and preventive treatments and their effect, and documentation of concomitant diseases. In case of a >10% discrepancy between the data collected during the visit—with the questionnaire and/or the direct interview—and the data reported in the database, the issue was discussed with the patient, who was then invited to consider the additional information before elaborating the final answer.

In this way, we used a hybrid methodology that combines cross-sectional data collected with the standardized questionnaire and during the actual study visit, with the review of data stored in our clinical database.

Our primary outcome was the difference in the number of female and male CH patients who satisfied ICHD-III criterion D for migraine-associated symptoms during their attacks. More specifically the criterion D was satisfied when “nausea and/or vomiting” or “photophobia and phonophobia” were present during CH attacks. Secondary analyses evaluated the difference in gender distribution of individual associated symptoms and as well as of all the other items included in the questionnaire and collected during the visit (see above).

### Statistical Analysis

The sample size was calculated with the Open Source Epidemiologic Statistics for Public Health (www.openepi.com). For the primary outcome we considered meaningful a difference between groups of at least a 20% based on previous reports and our clinical experience. The following parameters were used: two-sided confidence level: 95%; power 80%; ratio of sample size: 2; expected percent of male with outcome: 55%; expected percent of female with outcome: 75%; odds ratio: 2.43; risk/prevalence ratio: 1.36; risk/prevalence difference: 19.80. According to Fleiss method, the minimum suggested sample size was 228 (152 for male patients, and 76 for female patients).

For the statistical analysis, we used SPSS (Statistical Package for the Social Sciences) for Windows, version 21.0.

For quantitative variables the Kolmogorov-Smirnov test showed a normal distribution.

For quantitative variables, differences between females and males were tested with Student's *t*-test for unpaired samples. For categorical data, differences between females and males were examined with χ2 tests, or Fisher exact test where appropriate. Quantitative variables are presented as: mean ± standard deviation (95% confidence interval for mean). An alpha of 0.05 was used for all statistical tests.

## Results

We collected and analyzed data from 250 CH patients, 163 males (mean age 41.46 ± 10.37) and 87 females (mean age 42.24 ± 11.95), with a male to female ratio of 1.9:1. Most of our patients suffered from episodic CH (90.4 %) (Table 1).

**Table 1:** Demographic variables: comparison between sexes.

		**Male (M)**	**Female (F)**	***p*-value**
N		163	87	–
Age (years)		41.46 ± 10.37	42.24 ± 11.95	0.594
		(39.9–43.1)	(39.7–44.8)	
Type of CH (%)	Episodic	90.8%	89.7%	0.466
	Chronic	9.2%	10.3%	

*CH, Cluster Headache; M, Male; F, Female*.

**Figure 1:**
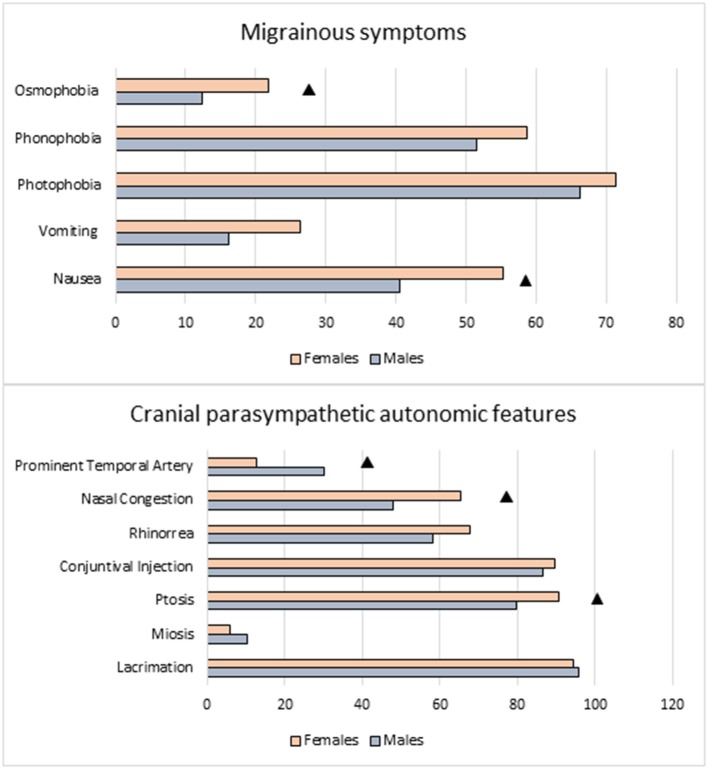
Migraine-like and trigeminal autonomic symptoms: comparison between sexes. ▲ Females vs. Males: *p* < 0.050.

We did not find statistically significant difference between male and female subjects in terms of satisfaction of ICHD-III criterion D. Indeed the criterion was satisfied by a quite high percentage of subjects in both sexes: F 73.6%, M 65.6%, *p* = 0.200. When we analyzed gender-related distribution of individual migraine-associated symptoms, we observed that nausea and osmophobia were reported more frequently by females than males: nausea F 55.2 vs. M 40.6%, *p* < 0.05; osmophobia F 21.8 vs. M 12.3%, *p* < 0.037. Vomiting, photo and phonofobia, were numerically more frequent in female patients, but the difference did not reach a statistically significant level (Table 2 and Figure 1).

**Table 2:** Clinical variables: comparison between sexes.

		**Female (F)**	**Male (M)**	***p*-value**
*N*		87	163	
CH onset (years)	Total	26.3 ± 12.7 (23.6–29.0)	27.9 ± 9.9 (26.4–29.4)	0.282
	Episodic	25.6 ± 12.7 (22.8–28.5)	27.3 ± 9.5 (25.8–28.9)	0.265
	Chronic	32.1 ± 11.8 (26.3–41.2)	33.5 ± 12.7 (26.5–40.5)	0.793
Family history of migraine (%)		68.6%	58.5%	0.119
Family history of cluster headache (%)		4.7%	9.1%	0.203
Bout frequency/year		1.03 ± 0.8 (0.9–1.2)	1.03 ± 0.7 (0.9–1.1)	0.957
Mean Duration of active phase (days)		43.4 ± 23.8 (37.9–48.8)	45.7 ± 29.6 (41.1–50.7)	0.553
Duration of attacks (min)		79.5 ± 48.9 (69.0–91.9)	64.5 ± 32.6 (60.9–71.6)	**0.004**
Attack frequency / day		2.28 ± 1.06 (2.0–2.5)	1.78 ± 0.98 (1.8–2.2)	0.053
**Distribution of migraine-associated symptoms**				
ICHD-III criterion satisfied		73.6%	65.6%	0.200
**Individual migraine-associated symptoms**				
Nausea		55.2%	40.6%	**0.048**
Vomiting		26.4%	18.2%	0.095
Photophobia		71.3%	66.3%	0.254
Phonophobia		58.6%	51.5%	0.174
Osmophobia		21.8%	12.3%	**0.037**
**Cranial parasympathetic autonomic features**				
Lacrimation		94.3%	95.7%	0.410
Miosis		5.7%	10.4%	0.156
Ptosis		90.8%	79.8%	**0.017**
Conjuntival injection		89.7%	86.5%	0.306
Rhinorrea		67.8%	58.3%	0.090
Nasal Congestion		65.5%	47.9%	**0.005**
Prominent temporal artery		12.6 %	30.1%	**0.001**

*CH, Cluster Headache; M, Male; F, Female*.

*In bold: significant p-values*.

As regards local autonomic symptoms, most of them were equally distributed in women and men, with the exception of ptosis and nasal congestion, which were more frequently reported in female sufferers: ptosis F 90.8 vs. M 79.8%, *p* = 0.017; nasal congestion F 65.5 vs. 47.9%, *p* = 0.005), and enlarged temporal artery, which was instead more frequently reported in males (F 12.6 vs. M 30.1%, *p* = 0.001) (Table 2 and Figure 1).

Pain location was typically orbital/retro-orbital in both sexes, without significant difference (*p* = 0.337), but women experienced a more widespread distribution of pain, as demonstrated by the higher percentage of female CH subjects who reported pain also in the zygomatic (F 25.3 vs. M 16%, *p* = 0.050), parietal (F 14.9 vs. M 7.4%, *p* = 0.049), and frontal (F 49.9 vs. M 36.8%, *p* = 0.037) areas.

CH women had a longer mean duration of untreated attacks than men (79.5 ± 48.9 vs. 64.5 ± 32.6 min, *p* = 0.004). We also detected a pattern toward a higher number of attacks/24 h in the female sex, which however did not reach a statistical significance (F 2.28 ± 1.06 vs. M 2.00 ± 0.98, *p* = 0.053).

The bout frequency was similar between sexes, with the majority of patients reporting only one per year. No gender differences were detected in the duration of bouts, which lasted 45.7 ± 29.6 days in men and 43.4 ± 23.8 days in women (*p* = 0.553) (Table 2).

A family history of migraine was quite frequent in both sexes, numerically more prevalent in women as compared to men (68.6 vs. 58.5%, *p* = 0.119). By contrast, the family history of CH was reported more frequently by male patients than females, but again the difference did not reach a statistically significant level (9.1 vs. 4.7%, *p* = 0.203). The mean age at CH onset was 27.9 ± 9.9 in men and 26.3 ± 12.7 in women (*p* = 0.282). Patients with episodic CH had a mean age at onset of 26.8 ±10 (27.3 in men and 25.7 in women), while those with the chronic form had a mean age at onset of 33.0 ± 12.1 (33.5 in men and 32.1 in women) (Table 1).

Interestingly, 61% of female patients reported occurrence of the onset of disease during periods of abrupt fluctuations of sexual hormones: 16 reported their onset of disease at menarche, 8 during the post-partum, 23 at the menopause, and 6 during the intake of birth control pills.

Concomitant thyroid diseases (F 23 vs. M 1.8%, *p* = 0.001) and psychiatric disorders, namely depression and anxiety (F 17.2 vs. M 9.2%, *p* = 0.04) were more frequent in women than men, while snoring and smoking habit were more frequent in men: M 53.4 vs. F 19.5% (*p* = 0.00) and M 67.5 vs. F 49.4% (*p* = 0.005), respectively.

## Discussion

CH is considered a predominant male disease, although several studies have pointed to a progressive decrease of the male-to-female ratio over time (2–5). This observation has stimulated, in recent years, speculations and investigations on the possible factors involved in this phenomenon and on the possible occurrence of differences in CH presentation between the sexes.

Available data on gender-related differences in CH are interesting but limited and partly discordant. This study provides additional information that overall suggests a relevant overlap of symptoms between migraine and CH, which is more marked in the female sex.

As regards our primary outcome, we did not find a significant difference in gender-related distribution of migraine-associated symptoms, as evaluated with the ICHD-III D criterion for migraine without aura. Indeed, the difference between sexes (8%) was lower than the value that we considered clinically meaningful defined (20%), but it seems worth noting that the criterion was satisfied in the large majority of CH subjects. Interesting findings were derived from our secondary analyses, which confirmed some previous data on the clinical presentation of CH and its gender differences, but also provided new pieces of information that are relevant for an improved understanding of CH pathophysiology.

### Associated Symptoms

In our study, women more frequently experienced nausea and osmophobia during cluster attacks. The presence of migraine-associated symptoms has already been reported in CH patients (17, 18), although in a lower percentage of patients than our population, but only a few studies have looked into gender differences. A previous Italian study noted an increased occurrence of nausea in CH women (10), Rozen et al. (8, 9) and Bahra et al. (5) reported an increased occurrence of nausea and vomiting in women with CH. The findings were not confirmed by Dong et al., whose population, however, included a very limited number of women (11).

Here, in a large and well-characterized clinical CH population followed at a tertiary referral center, we confirm the presence of nausea as a distinctive gender-related sign in female CH. In addition, we report a higher incidence of osmophobia in female CH subjects, which, to the best of our knowledge, has never been investigated in this detail so far. Osmophobia has a high specificity for migraine (19) and has been proposed as an additional feature for migraine diagnosis. Our finding regarding osmophobia may thus reinforce the hypothesis that CH and migraine shares some pathophysiological mechanisms, especially in the female sex.

In our population, ptosis and nasal congestion were more frequently reported in women, while an enlarged temporal artery was predominant in males. Published data on a gender-related differential expression of autonomic symptoms are inconclusive. Rozen et al. (8) reported a tendency toward a higher prevalence of ptosis and miosis in CH women. In a more recent study, other Authors confirmed the increased occurrence of ptosis in CH women, together with a higher occurrence of eyelid edema (4). Male CH subjects seem to experience more frequently than women lacrimation and facial sweating (9, 20). In partial agreement with our findings, another Italian group noted a lower occurrence of ptosis, tearing and nasal congestion in a subgroup of males with late onset of CH (12), to suggest a possible role of age on the differential expression of associated symptoms.

### Duration of CH Attacks

We confirmed that women had a significantly longer duration of untreated CH attacks than men, as previously noted by Kudrow (21). This is partially in contrast with others studies (8–10) that reported a tendency toward a shorter attack duration in females together with a similar daily attack frequency. These contradictory findings may reflect recall biases, even more so in retrospective studies using questionnaires mailed to patients. In line with this, a recent Danish study that compared retrospective and prospective descriptions of attack features found that, when compared to men, women often report longer and more severe attacks with more severe migrainous symptoms (22). In our study, we collected cross-sectional data from an ongoing bout, that were matched with the data recorded over time in our hospital database from patients that were regularly followed at our headache center and were used to fill in a headache diary during their active bouts. The weekly reports of these diaries are recorded and stored in our clinical database. Altogether, we believe that our hybrid methodology provides a considerable degree of robustness to the data collected, while minimizing as much as possible the occurrence of recall biases and the variability between different bouts.

### Pain Location and Extension

In our study both sexes reported the classic pain location within the distribution territory of trigeminal V1 (orbital or retro-orbital areas), but we noted that women more frequently reported a higher widespread distribution of pain that extended over the zygomatic, parietal and frontal regions. This is consistent with previous reports of a frequent location of pain outside V1 trigeminal area in CH women (5–9). Furthermore, in line with our findings, in a very recent Korean study, focused on the assessment of clinical gender characteristics in subjects with CH in a prospective registry, women more frequently experienced pain in the forehead, compared to men (46.3 vs. 30.1%, *P* = 0.043) (20).

### CH Onset

The mean age at CH onset was similar in both sexes, even though we observed a trend toward an earlier occurrence in female subjects, as reported in literature (8, 23–25). More importantly, we report that a non-negligible percentage of CH women associate the onset of their disease with reproductive events of their life, such as menopause, menarche, pregnancy, or post-partum and hormonal contraceptives (in order of prevalence), thus suggesting a possible role for important hormonal shifts in the pathogenesis of CH.

Previous studies looked at the fluctuations in the prevalence of the sex distribution across ages: Kudrow reported an increased frequency of CH in women when they reached the age of fifty or sixty (2), and Ebkom and Mosek confirmed an initial onset of CH after the menopause (14, 26). This finding has been recently confirmed, mostly for the chronic form of CH, by Manzoni et al. (27), who also noted an increased occurrence of CH in women before the age of 14. These observations fit well with our findings regarding the role of hormones in CH women when considering that several studies have reported a tendency toward a bimodal distribution of age at onset of CH in women (2nd−3rd decade and 5th−6th decade) (8, 25), while CH onset in men manifests peaks during the 3rd decade (8).

In a large population of CH patients compared with migraine females, van Vliet et al. (15) found that menstruation, use of oral contraceptive, pregnancy, and menopause had a much smaller influence on CH attacks than on migraine, as reported by other earlier studies (2, 10). Therefore, unlike migraine, no definitive relationship between CH and female reproductive phases of life could be established in a recent review of the literature (16). Hence, the importance of our present findings to lend further evidence on the existence of a hormonal link between CH female population and disease onset, could stimulate further studies, possibly prospective, to better evaluate the role of hormonal changes in CH pathophysiology.

### Comorbidities—Associated Conditions

A body of literature has connected psychiatric comorbidities, especially depression, anxiety, and aggressive behavior to CH patients, without any gender differences (28, 29). Whether psychiatric comorbidities in CH is the consequence of the psychological effect of the extreme intensity of the attacks or it represents a manifestation of a common pathophysiological process is still a matter for debate. The increased prevalence of depression and anxiety observed in our female population is in line with a previous large internet American survey (9) and with a very recent Korean study (20), although we cannot rule out the possibility that the higher incidence may be simply related to the higher epidemiological impact of psychiatric conditions in the female sex. More disease-specific seems the increased prevalence of thyroid disease in our female population, when considering that the prevalence of thyroid disorders in the Italian population is lower: 10% according to the official data from www.portaledellasalute.it. It is interesting to observe that thyroid disease is associated to poorer response to standard treatments for mood disorders (30). To the best of our knowledge, our study is the first to ever report this triple connection CH-depression-thyroid disease in CH females. Though available evidence does not warrant any pathophysiological speculations at this moment, it seems nonetheless important to consider both these comorbidities when deciding the choice of the preventative treatment in women suffering from CH.

The higher occurrence of snoring and smoking in men is in line with previous results (7, 31–33). Smoking prevalence has been consistently reported to be significantly higher in CH patients, compared to general populations (48–68%), and also when stratifying by sex (34); this close relation between smoking habit and cluster headache has been identified as a contributing factor of the disease in predisposed individuals.

## Conclusion

We confirmed the presence of distinct gender-related differences in CH and added some novel information that may be relevant to advance our knowledge of the pathophysiological mechanisms underlying the disease, to improve the diagnostic process and possibly lead to an improved management of CH.

## Data Availability Statement

The datasets generated for this study are available on request to the corresponding author.

## Ethics Statement

The studies involving human participants were reviewed and approved by Ethics Committee of IRCCS San Raffaele Scientific Institute, Milan, Italy. The patients/participants provided their written informed consent to participate in this study.

## Author Contributions

MA and CT designed the project. MA wrote, reviewed, and edited the article. LA and AP collected data from the questionnaire and the database. MA, RD, EP, and GS enrolled patients. RD and RG performed statistical analysis. CT reviewed and revised the manuscript. All authors contributed to the planning and development of the study, supervised by MA. All authors read and approved the final manuscript.

### Conflict of Interest

The authors declare that the research was conducted in the absence of any commercial or financial relationships that could be construed as a potential conflict of interest.
